# To the Editors of the Medical and Physical Journal

**Published:** 1805-05-01

**Authors:** 


### To the Editors of the Medical and Physical Journal.

"Suum cuique."

GENTLEMEN,

THERE is no art or science, I believe, in which so frequent contentions have taken place respecting discoveries, so much animosity and ignorance displayed in their controversies, or such injustice done in conferring the honors due to merit, as in our profession. Teachers and men of eminence oftentimes not content with that praise which is justly bestowed on them, are ambitious of immortalizing their fame by some great discovery or improvement; and are not ashamed to steal the laurels from the brow of one to whom they are indebted, and impiously endeavour to crown themselves with the pilfered honours. Knowing that nothing imposes on the world so much as modesty, they claim no merit to themselves, "their discovery was the effect of accident," or from the improvement being of extensively beneficial consequence, it could only be "by inspiration." Their characters being established, the justice of their claims is admitted by the ignorant part of the profession, who never fail in hailing that person as the discoverer, who first informed them of it. Their works being copied and quoted, impose on men of superior abilities, who are always desirous to bestow applause where they think it justly due. Thus their claims to discoveries and improvements, to which they have not the slightest right, receive the united sanction of time and ignorance. Hence we have the *foramen Monroianum* and *antrum Highmorianum*; and, what led me to make these remarks, the introduction of the ligature attributed to Ambrose Parée. To this author has this improvement always been ascribed by the French surgeons, from that national vanity which induces them to claim for their countrymen every discovery and improvement in every art and science. A very voluminous work, wherein the subject of diseased and wounded arteries is most voluminously treated, has caused Parée's claim to be pretty generally acknowledged in this country. That this author should adopt this error, I am not surprized, as his writings prove him very conversant with the works of other French surgeons besides Parée. But my astonishment was excited, on seeing it supported by Mr. Simmons of Manchester, in a very excellent paper in your Journal, on Amputation. I can hardly suppose Mr. S.

 unacquainted unacquainted with the writings of Celsus, the Father of Surgery; for fear he should have forgot it, I shall quote a passage, which will completely refute the claim of Parée to the merit of introducing the ligature. In his 26th Chapter, Lib. iii. entitled "Curatio adversus Profusionem Sanguinis in Vulneribus," after mentioning dry linen, sponges squeezed in cold water, linen moistened with vinegar, and caustic medicines; he says, if these should not succeed, we must effect the same thing by milder means. "Sed, si semel ad ea decurritur, potius quæ mitius idem efficiunt," which he thus describes, "Quod si illa quoque profluvio vincuntur; venæ quæ sanguinem fundunt, apprehendendæ circaque id, quod ictum est, duobus locis deligandæ intercidendæque sunt, ut et in se ipsæ coeant, et nihilominus ora præclusa habeant." Nor is Celsus the only author who mentions it. Paulus Ægineta not only used it in wounded arteries, but practised it in cases of aneurism, in the same manner as it is done at present. That Parée was acquainted with these authors I will not assert; but thus much may be safely averred, that the Italian surgeons, with whom Parée had such frequent intercourse in the different incursions of Henry IV. the greatest and wisest of monarchs, into Italy, were ignorant neither of their opinions or their practice; and it is more than probable that Parée learnt it from them. The ligature is also mentioned by Rufus and several other writers of antiquity.

CHIRURGICUS.

